# Shelter for the Homeless

**Published:** 1897-06-05

**Authors:** 


### Shelter for the Homeless.

A report recently made by the medical officer to the London County Council on the sanitary condition of the shelters for the homeless poor in London shows how necessary it is that these institutions should be brought under the control either of the Home Office or of the sanitary authorities.

The present condition of affairs is anomalous in the extreme, and is fraught with no small danger to the public health. We hear a great deal just now about the crowds of more or less wealthy strangers who are flocking to London to take their share in the coming Jubilee celebrations, and every day we see evidence that London is likely to be for the next month a place of pilgrimage where much money will be spent.

The other side of the shield is not so obvious, but we all know that it is there, and that London will at the same time be the loadstone which will attract all the impecunious wasters, all the loafers and ne'er-do-wells, all the tramps and hangers-on, and even all the honest workers seeking for employment who can scrape together enough money to land themselves in such a temporary Eldorado. All these people hope to get a picking out of the money which they hear will be flying about so freely—yet nine out of every ten will be disappointed. Huddled together on the Embankment, stretched on the damp grass in the parks, crowding the doss-houses, and filling the casual wards, the disappointed candidates for stray coin will be found night after night, and we may be sure that the shelters will be full.

Nothing then could well be more appropriate to the occasion than this report in which Dr. Young, who has recently inspected all these shelters, sets forth the sanitary or rather the insanitary conditions which prevail within them. Here we have at last the definite data which have been so long refused; the cubic space, the character of the sleeping accommodation and of the sanitary appliances, the condition of the rooms by day, and their condition by night; and as the outcome of it all we find that the accommodation provided in about two-thirds of these shelters is such as would not be tolerated in the lowest common lodging house in the metropolis.

In view of this condition of affairs the question which has been asked so many times before crops up again, viz., Why should people acting under the cloak of charity be allowed to do things which would be put down with a firm hand by the law if done by anyone else?

No doubt people will be found ready to say that such shelter as is given is at least better than nothing. But such an argument is begging the whole question. The Common Lodging Houses Acts were passed not for the sake of the lodger but of the public; not for the sake of ensuring that the dosser got his due, but of preventing a state of overcrowding which was opposed to the public safety; and Mr. Shirley Murphy, in reporting on these shelters to the Council, very properly says that the fact that they are not carried on for profit is no reason why they should be exempted from the requirement that their dormitories should not be overcrowded. He maintains that the conditions of life of no human being ought to be allowed to sink below a certain minimum standard; that when this principle is infringed there is not only danger to the individual but to the community, and that the State has made provision available for those who are unable to procure this minimum for themselves.

This really puts the matter in a nutshell. Except in the fact that the shelters are ostensibly charitable institutions, there is no difference between them and common lodging-houses, and we entirely fail to see why they should not be placed under the same legal control. It is a matter of some little interest to note that, when Lord Coleridge delivered the judgment on the strength of which these shelters remain independent of all inspection and control, he gave as his reason that the shelters, being charitable institutions, would not be liable to overcrowding such as he thought might not unnaturally occur where lodgings were let as a commercial undertaking, an overcrowding which he admitted might be productive of great disaster to the community. But now we know that this overcrowding does actually occur; and so we are in the absurd position of being tied down by a legal decision, although we know all the time that in giving it the judge acted under a misapprehension! Such is law.

